# Time-Resolved FDTD and Experimental FTIR Study of Gold Micropatch Arrays for Wavelength-Selective Mid-Infrared Optical Coupling

**DOI:** 10.3390/s21155203

**Published:** 2021-07-31

**Authors:** Ying Fu, Tom Yager, George Chikvaidze, Srinivasan Iyer, Qin Wang

**Affiliations:** 1 School of Information Technology, Halmstad University, SE-301 18 Halmstad, Sweden; 2 Institute of Solid State Physics, University of Latvia, LV-1063 Riga, Latvia; tom.yager@cfi.lu.lv (T.Y.); georg.chikvaidze@cfi.lu.lv (G.C.); 3 Senseair AB, SE-824 08 Delsbo, Sweden; sri.iyer@senseair.com; 4 RISE Research Institutes of Sweden AB, Box 1070, SE-164 25 Kista, Sweden

**Keywords:** FDTD, FTIR, metal micropatch arrays, near field optics, far field optics, nano fabrication, electron beam lithography, infrared sensing

## Abstract

Infrared radiation reflection and transmission of a single layer of gold micropatch two-dimensional arrays, of patch length ∼1.0 μm and width ∼0.2 μm, have been carefully studied by a finite-difference time-domain (FDTD) method, and Fourier-transform infrared spectroscopy (FTIR). Through precision design of the micropatch array structure geometry, we achieve a significantly enhanced reflectance (85%), a substantial diffraction (10%), and a much reduced transmittance (5%) for an array of only 15% surface metal coverage. This results in an efficient far-field optical coupling with promising practical implications for efficient mid-infrared photodetectors. Most importantly we find that the propagating electromagnetic fields are transiently concentrated around the gold micropatch array in a time duration of tens of ns, providing us with a novel efficient near-field optical coupling.

## 1. Introduction

The interaction between light and microstructured materials offers great advantages for a wide range of applications including optoelectronics [1,2], bioimagings [3,4] as well as space exploitation, e.g., Laser Interferometer Gravitational-Wave Observatory (LIGO) where radiations of 30 Hz∼7.0 kHz are used to study cosmic gravitational waves [5]. Infrared radiation (IR) photodetectors have been under continuous research and development where new innovations are a constant demand, especially from industrial perspectives regarding cost effective IR products with enhanced performance [6,7]. However, the wide dissemination of these IR photodetectors is still limited due to a lack of solutions for high sensitivity, wavelength-selectivity, room-temperature operation, miniaturization and low-cost.

One key aspect is to enhance optical coupling with proper wavelength selectivity between the incident radiation and the photon-absorbing electrons [8,9]. Subwavelength hole array perforated in metal thin film was shown to significantly enhance light absorption [8,10,11], whilst three-dimensional microstructure gratings [12] and synchronously wired infrared antennas [13] have also been designed and fabricated to demonstrate improved IR absorption.

Metal microstrip arrays have been receiving an increasing attention because of their response-wavelength selectivity based on structure geometry [14,15,16,17], particularly for IR sensing and imaging applications [18,19,20,21]. IR detectors utilizing nano-micro metal-array plasmonic metamaterials hybrid with graphene have been demonstrated recently at mid-IR wavelength regime [22,23].

The design and development of high performance IR sensors can greatly benefit from quantitative analysis of the time-resolved electromagnetic field development in space. Significantly enhanced light absorption, mostly in the visible and near infrared (NIR) spectral ranges, through hole array perforated in metal thin film was initially observed and reported in 1998 [24]. Many theoretical studies were subsequently performed and reported [25,26,27,28], while the energy transport mechanism was only clearly unraveled by meticulous time-resolved study [29].

Most recent reports have studied rather complex systems, e.g., metal-insulator- metal [14], and graphene hybridized devices [22,23].

In this work, we combine computational simulations with experimental characterizations of nanofabricated single layers of two-dimensional gold micropatch arrays to understand and design wavelength-selective far- and near-field infrared optical couplings that operate in mid-IR wavelength regime aiming for, e.g., CO${}_{2}$ and alcohol sensing [30].

## 2. Materials and Methods

**Figure 1:**
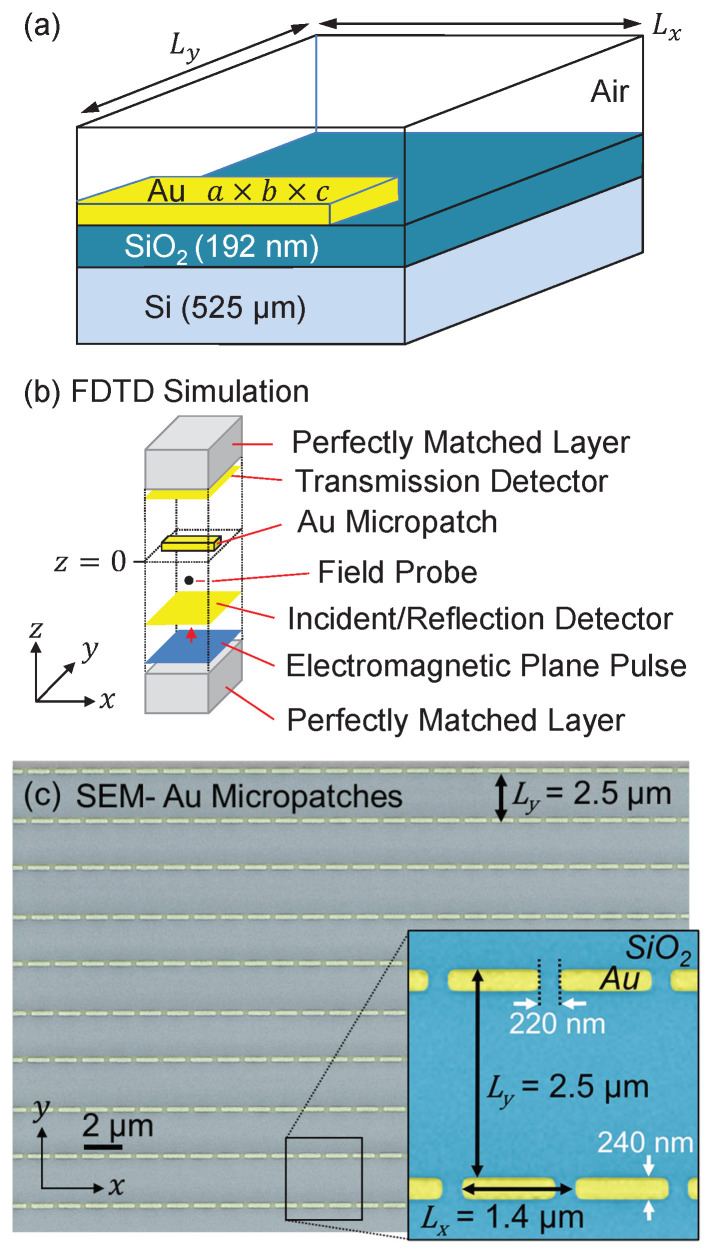
(**a**) Schematic illustration of a unit cell of the gold micropatch array, on the surface of a SiO${}_{2}$/Si substrate. (**b**) Configuration of the FDTD study. (**c**) SEM images of an experimentally fabricated Au/Ti micropatch array (colourised for clarity) (a=1.18 μm, b=0.24 μm, c=50 nm, $L_{x}=1.4$ μm and $L_{y}=2.5$ μm), total array area =2×2 mm${}^{2}$.

Figure 1 shows an example of the two-dimensional gold micropatch array on the surface of an an insulating SiO${}_{2}$/Si substrate. The size of the metal patch is denoted as a×b×c, where *a* is the length in the *x* direction, *b* is the width in the *y* direction, and *c* is the thickness in the *z* direction. In this study, a=1.0∼1.8 μm, b=0.2∼0.28 μm, and c=50 nm. The periods of the array are denoted as $L_{x}$ and $L_{y}$ in the *x* and *y* direction, respectively.

For our finite-difference time-domain (FDTD) study, a spatial resolution down to 1.0 nm is required in order to resolve the electromagnetic field distribution in Au micropatches of thickness of c=50 nm (the *z* dimension of the micropatches), while the periods of the micropatch arrays in the *x* and *y* dimensions are about 1∼2 μm; Moreover, the spatial extension in the *z* dimension of the calculation domain must be large (∼10 μm) for studying far fields. All these require a huge computer memory. Another significant challenge for the FDTD study is about the temporal resolution, which is the main focus of the present study. The applied FDTD code was developed in-house for many studies [8,29]. For the present study, our in-house FDTD code uses up to 200 GB RAM (when studying Au micropatch arrays of $L_{y}=2.5$ μm) in a mini-supercomputer Intel(R) Xeon(R) 144 cores, 500 GB RAM, 20 TB HDD.

Dielectric coefficients of gold are numerically described using Lorentz equation with two poles by fitting refractive index and extinction coefficient data (wavelength 1∼10 μm) obtained from [31]. Multiple probes and detectors were used to study the time-resolved transmission, reflection and diffraction of a normal-incident electromagnetic plane pulse, of either $\left(E_{x},H_{y}\right)$, or $\left(E_{y},H_{x}\right)$ polarization, centered at the frequency 0.52 μm${}^{-1}$ (wavelength 1.923 μm), with a frequency half bandwidth 0.60 μm${}^{-1}$, as schematically described in Figure 1b. Periodic boundary conditions were applied in the *x* and *y* directions, while the ends of the calculation domain in the *z* dimension were simulated by perfectly matched layers (PMLs). Time step duration of FDTD calculation was $3.659\times 10^{-12}$ s, while the spatial step length was set to 1.0 nm. The total number of simulated time steps was $6\times 10^{4}$, long enough that all fields propagated away from the space domain of the FDTD study.

To validate our FDTD study and demonstrate optical coupling, corresponding periodic micropatch arrays were fabricated by electron beam lithography and optically characterized by Fourier transform infrared spectroscopy (FTIR). The micropatch arrays were defined with a Raith eLine Plus electron beam lithography system (Raith, Dortmund, Germany) using PMMA resist and standard lift-off processing. A SiO${}_{2}$ surface, thickness 192 nm, was thermally grown by standard PECVD on commercially available double-side-polished silicon wafer (MicroChemicals, Ulm, Germany) (thickness 525 μm). Gold films (∼45 nm), with a thin titanium adhesion nanolayer (∼5 nm) were deposited on the SiO${}_{2}$ surface in an Edwards Auto 306 thermal evaporator (Edwards, Burgess Hill, UK) (vacuum pressure $10^{-6}$ mBar). A total of nine metal micropatch arrays were studied, fabricated across two different SiO${}_{2}$/Si wafers. Each array was of uniform 2×2 mm${}^{2}$ dimensions, and spatially separated by at least 1 mm, to allow reliable identification by FTIR.

Following fabrication, the surface geometries were measured and analysed by scanning electron microscopy (SEM), using the same eLine system. Micropatch dimensions were determined with an approximate measurement uncertainty of ±0.025 μm, originating from geometric non-uniformity of fabricated metal micropatches and SEM measurement precision. Both the array-to-array and wafer-to-wafer repeatability was found to be within this same uncertainty range. Metal thicknesses were verified with a Dektak 150 Surface Profiler (Veeco, Santa Barbara, CA, USA) within ±2 nm. Figure 1c shows SEM pictures of 1.18×0.2×0.05 μm${}^{3}$ gold micropatch array ($L_{x}=1.4$ and $L_{y}=2.5$ μm).

Micro reflection and transmission Fourier-transform infrared spectroscopy (FTIR) measurements were performed in ambient air by a VERTEX 80v FT-IR spectrometer (Bruker, Ettlingen, Germany) attached to a HYPERION 2000 FT-IR microscope (Bruker, Ettlingen, Germany), where the FTIR photodetector was positioned to detect directly either the transmission or the reflection light. The FTIR spectra were recorded in the range of 500∼5000 cm${}^{-1}$ (2∼20 μm), with a resolution of ±2 cm${}^{-1}$, measured over a 100×100 μm${}^{2}$ area.

## 3. Transmittance, Reflectance, and Diffractance

Au micropatch arrays on SiO${}_{2}$/Si were carefully designed by FDTD study and experimentally verified by FTIR investigation of EBL fabricated device structures.

Initial FDTD study showed that the interaction of IR radiation with the Au/Ti microstrip was dominated by the thick Au layer (45 nm) whilst the thin Ti surface adhesion layer, only 5 nm, was found to be too thin to interact with IR radiation of wavelength of 4 μm. Moreover, since our Au micropatches were formed by low-temperature evaporation and lift-off, i.e., without any high temperature processes, it is believed that the interdiffusion effect of Au-Ti in our samples is insignificant. Finally, FTIR spectra measured repeatedly with weeks of time intervals were identical. We did not include the interdiffusion effect of Au-Ti in our FDTD study. Subsequent FDTD study focused on pure Au micropatch arrays.

**Figure 2:**
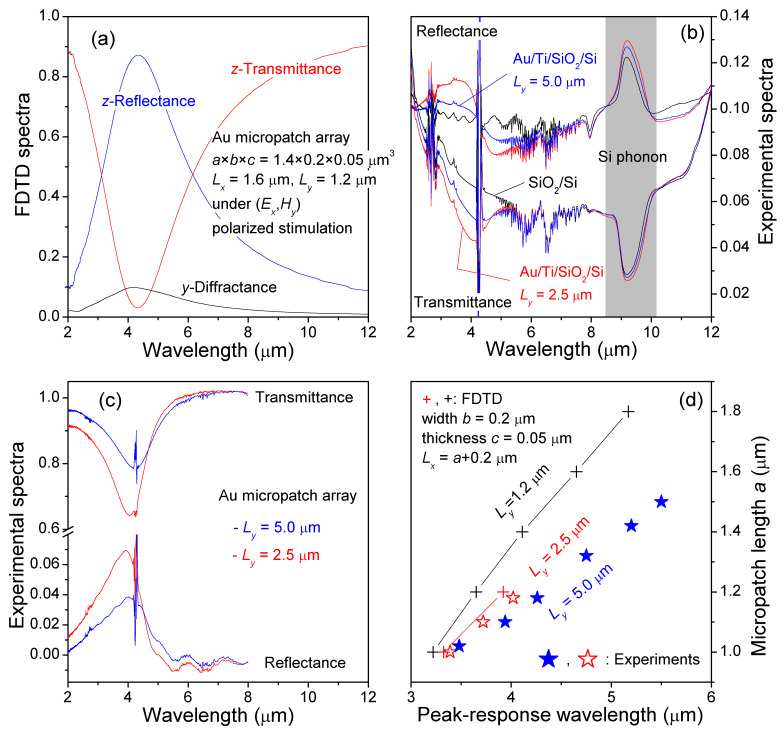
FDTD and FTIR studies. (**a**) FDTD transmittance, reflectance, and diffractance of one Au micropatch array 1.4×0.2×0.05 μm${}^{3}$ under $\left(E_{x},H_{y}\right)$ polarized stimulation. (**b**) FTIR transmittance and reflectance spectra of SiO${}_{2}$/Si and two Au/Ti/SiO${}_{2}$/Si samples. Blue: 1.18×0.24×0.05 μm${}^{3}$, $L_{x}=1.2$ μm, $L_{y}=5.0$ μm; Red: 1.18×0.24×0.05 μm${}^{3}$, $L_{x}=1.1$μm, $L_{y}=2.5$ μm. The upper three spectra are reflectance, while the lower three are transmittance spectra. (**c**) Extracted transmittance and reflectance spectra of the Au/Ti micropatch arrays. (**d**) Peak-response wavelength as a function of the Au micropatch length *a*.

By integrating energy fluxes over a total time span of $6\times 10^{4}$ time steps, the calculated transmittance, reflectance, and diffractance of one Au micropatch array, a×b×c=1.4×0.2×0.05 μm${}^{3}$ and $L_{x}\times L_{y}=1.6\times 1.2$ μm${}^{2}$, are presented in Figure 2a. At peak-response wavelength of 4.2 μm, they reveal that the initial incident energy flux, of $\left(E_{x},H_{y}\right)$ polarized stimulation, along the *z* axis was approximately 85% reflected, 5% transmitted, and the remaining 10% diffracted into the *y* direction. No perceptible loss was observed.

The 85% reflectance and only 5% transmittance are extraordinary in terms of what can be expected by light ray penetration through the Au micropatches with a surface metal coverage of

$$\mathrm{Surface}\;\mathrm{metal}\;\mathrm{coverage}=\frac{a\times b}{L_{x}\times L_{y}}=\frac{1.4\times 0.2}{1.6\times 1.2}=14.6\%$$

Extraordinary transmission, i.e., the opposite phenomenon shown in Figure 2a, was previously studied and reported in a subwavelength hole array in a thin metal film [29].

These extraordinary transmittance and reflectance are the results of a strongly modified photonic dispersion created by the periodic array of Au micropatches. Ref. [32] shows that when excitons in semiconductor quantum dots (QDs) coupled with the incident radiation, they modify the dielectric coefficients of the QDs, resulting in a photonic crystal with a strongly modified photonic dispersion when QDs are placed periodically in space. With properly designed geometry of the QD photonic crystal, the reflectance of a single plane containing periodic arrays of QDs can reach 100% [33]. Similar photonic dispersion is expected to be created by our micropatch array, resulting in the observed extraordinary transmittance and reflectance, albeit in different responsivities and response wavelengths.

Our FDTD study of Au micropatch arrays showed that the $\left(E_{y},H_{x}\right)$ polarized stimulation light transmitted through our Au micropatch array without significant impediment.

Experimental unpolarized FTIR spectra of SiO${}_{2}$/Si, denoted as transmittance $t_{2}$ and reflectance $r_{2}$, and Au/Ti micropatch array on SiO${}_{2}$/Si, *T* and *R*, are shown in Figure 2b. Since the FTIR spectra were acquired in ambient air, spectral lines of various gas molecules are clearly observed in the figure.

The extraction of the transmittance $t_{1}$ and reflectance $r_{1}$ spectra of the Au/Ti micropatch array was performed as follows. Following light reflection and transmission from Au/Ti micropatch array then to SiO${}_{2}$/Si, we included multiple reflections and transmissions between Au/Ti micropatch array and SiO${}_{2}$/Si so the total transmittance and reflectance are

(1) $$\begin{array}{cc} T & =t_{1}t_{2}+t_{1}r_{2}r_{1}t_{2}+t_{1}r_{2}r_{1}r_{2}r_{1}t_{2}+\ldots \\ \; & =\frac{t_{1}t_{2}}{1-r_{2}r_{1}}\\ R & =r_{1}+t_{1}r_{2}t_{1}t_{2}+t_{1}r_{2}r_{1}r_{2}t_{1}+t_{1}r_{2}r_{1}r_{2}r_{1}r_{2}t_{1}+\ldots \\ \; & =r_{1}+\frac{t_{1}^{2}r_{2}}{1-r_{2}r_{1}} \end{array}$$

respectively, from which we obtain

(2) $$t_{1}=\frac{\frac{T}{t_{2}}\left(1-Rr_{2}\right)}{1-\frac{T^{2}}{t_{2}^{2}}\;r_{2}^{2}}\;\;\;,\;\;\;r_{1}=\frac{R-\frac{T^{2}}{t_{2}^{2}}\;r_{2}}{1-\frac{T^{2}}{t_{2}^{2}}\;r_{2}^{2}}$$

Extracted transmittance and reflectance of the Au/Ti micropatch array are presented in Figure 2c. The direct comparison between Figure 2a,c shows close agreement between theory and experiment.

- FDTD results of Figure 2a are for $L_{y}=1.2$ μm under one polarized stimulation of $\left(E_{x},H_{y}\right)$. Since there is no reflection but perfect transmission for $\left(E_{y},H_{x}\right)$ polarization, the reflectance and transmittance values for unpolarized stimulation at 4.2 μm are expected to be 42% and 10%, respectively. Experimental Figure 2c are for $L_{y}=5.0$ μm (80%, 4%) and $L_{y}=2.5$ (60%, 7%) under unpolarized stimulation. When we extrapolate the peak response values to $L_{y}=1.2$ μm, we would get approximately 40% reflectance and 10% transmittance, respectively, very close to the FDTD results.
- Figure 2d shows the consistent theory-experiment relationship between $L_{y}$ and the peak-response wavelength. Note that the experimental peak response wavelength was limited to an uncertainty of up to ±0.15 μm by the FTIR peak broadening and spectral noise. $L_{y}$ in FDTD study was relatively small due to limited computation memory size.
  For our micropatch array designs, we expect that the most crucial variable is the metal micropatch geometry. For each fabricated array, the micropatch geometries were measured by SEM, with a geometric repeatability across fabrication runs of approximately 10∼20 nm. For the metallization itself, the materials and methods used are also relatively simple and also quite well controlled.
  The approximate measurement uncertainties, relevant to Figure 2d, are: (a) Peak-response wavelength, ±0.15 μm. This is limited by the FTIR peak broadening and spectral noise. (b) Micropatch length, ±0.025 μm. This is limited by geometric nonuniformity of the patches and SEM measurement uncertainty.

In addition to the geometry-dependent response peak at wavelength 4.2 μm, a second reflection and transmission band at about 9∼9.5 μm was observed in the experimental FTIR spectra in Figure 2b. This relates to the optical emission and absorption of Si phonons (see further discussion below).

Having thus verified our FDTD model, we now proceed to study the dynamics of the enhanced reflectance and reduced transmittance.

## 4. Time-Resolved FDTD Study

**Figure 3:**
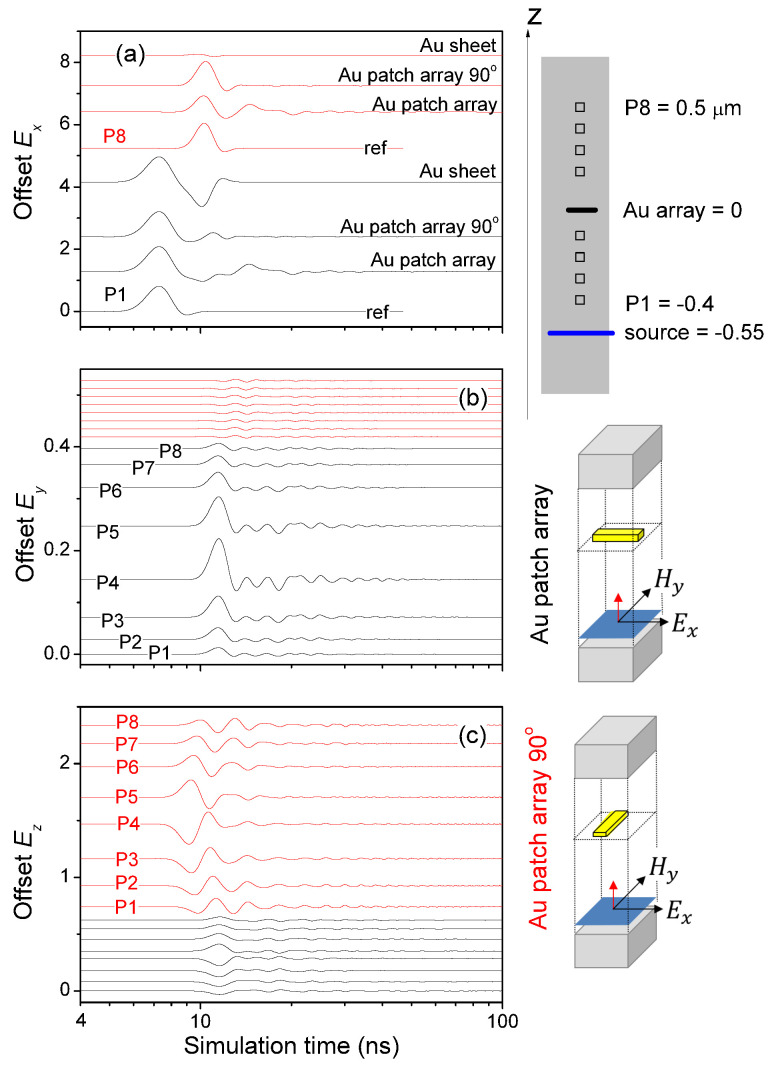
Temporal developments of the electric field components (**a**) $E_{x}$ detected at P1 (black lines) and P8 (red lines), (**b**) $E_{y}$ and (**c**) $E_{z}$ at the 8 probes (P1, P2, *…*, P8) under $\left(E_{x},H_{y}\right)$ polarization through different microstructures, simulated by FDTD.

The transient dynamics of the Au micropatch arrays was investigated by time-resolved FDTD study. For clear, consistent numerical presentations, in the following we designate $\left(E_{y},H_{x}\right)$ polarized stimulation of Au micropatch array by rotating Au micropatch array $90^{\circ }$ in the xy plane under $\left(E_{x},H_{y}\right)$ polarized stimulation. Referring to Figure 3, the initial incident pulse $\left(E_{x},H_{y}\right)$ has only nonzero $E_{x}$ and $H_{y}$. The temporal development of this incident pulse was to be monitored by 8 probes denoted as P1, P2, *…*, P8, positioned at z=−0.4,−0.3,−0.2,−0.1,0.2,0.3,0.4,0.5 μm. The source was positioned at z=−0.55 μm, with the Au micropatch array located at z=0.

Four microstructures were studied. “ref” was just the light propagation through vacuum. When an Au micropatch array was introduced, the propagating pulse was significantly modified, see Figure 3a, predominantly the diffracted $E_{y}$ shown in Figure 3b. The back-and-forth diffraction between $E_{x}$ and $E_{y}$ remained for a very long time around the Au micropatch array, see the amplitude variations of $E_{y}$ in the 8 probes. This caused the strong reflection. Most critically, Figure 3b shows that the strong reflection of the $\left(E_{x},H_{y}\right)$ field by the Au micropatch array occurred via the diffraction between $E_{x}∼E_{y}$ in a time duration in the order of tens of ns.

When we rotated the Au micropatch array $90^{\circ }$ to study the other orthogonal polarization, we observed similar but much weaker $E_{x}∼E_{y}$ diffraction, see red lines in Figure 3b. Instead, $E_{x}∼E_{z}$ diffraction was much stronger here in space and quicker in time, see red lines in Figure 3c, resulting in the perfect transmittance of $\left(E_{y},H_{x}\right)$ polarized stimulation mentioned in the previous section. Similar to the strong refection $\left(E_{x},H_{y}\right)$ field by the Au micropatch array, Figure 3c shows that the perfect transmittance through the Au micropatch array $90^{\circ }$ is not direct; It is intermediated by $E_{x}∼E_{z}$ diffraction in a time duration in the order of tens of ns.

**Figure 4:**
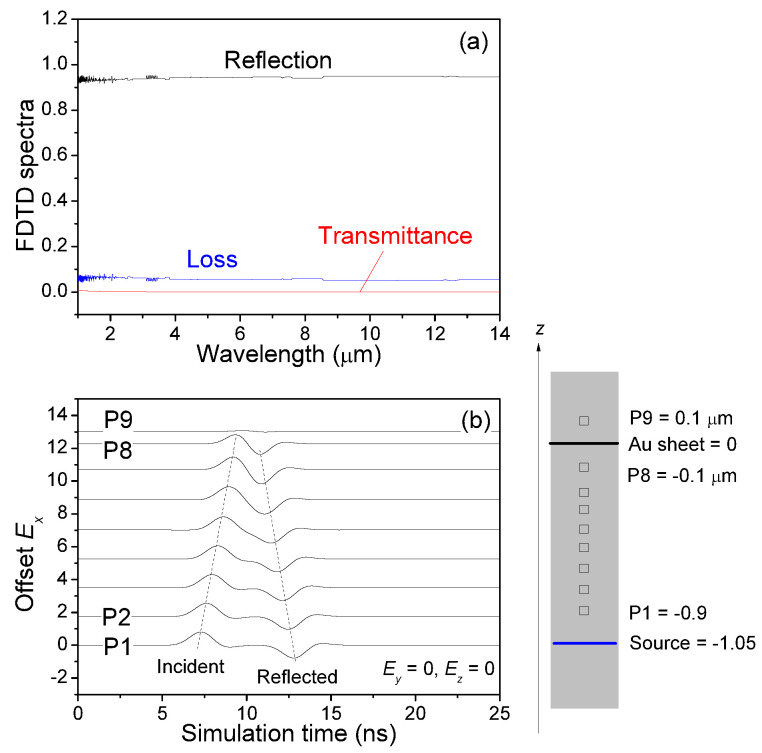
(**a**) Simulated reflection, transmittance, and loss of a perfect Au sheet with a thickness of 50 nm. (**b**) Temporal developments of the electric field component $E_{x}$. During the whole simulation time, $E_{y}=E_{z}=0$, at the 9 probes for initial incident $\left(E_{x},H_{y}\right)$ polarization.

We further studied a perfect Au sheet in which we observed a direct reflection of approximately 92%, very small transmission, virtually no diffraction at all, and a significant loss (ca 8% for the 50 nm thick Au sheet), see Figure 4, for the IR radiation of wavelengths of our interest. The small thickness of the Au sheet allowed a weak $E_{x}$ field detected at the ninth probe located at the other side of the source.

The most important phenomenon revealed in Figure 3 is the back-and-forth diffractions of $E_{x}∼E_{y}$ and $E_{x}∼E_{z}$ in both space and time, which is the results of a strongly modified photonic dispersion created by the periodic array of micropatches [32]. Similar temporal diffractions of the *H* field, $H_{y}∼H_{x}$ and $H_{y}∼H_{z}$, were revealed in our FDTD study. The primary incident $E_{x}$ field and the diffracted $H_{z}$ field resulted in the *y*-direction energy flux shown as the blue curve in Figure 2a. There was also a diffracted energy flux in the *x* direction, which, however, was much weaker.

Furthermore, the energy of the incident light at wavelength around 4.2 μm was strongly diffracted by the Au micropatch array in a spatial region of approximately ±0.3 μm above and below the micropatch array plane, see Figure 3b, until the $E_{x}$ field eventually propagated away in the ±z directions, from approximately $t=3\times 10^{3}$ to $t=10^{4}$ (time step length = $3.659\times 10^{-12}$ s).

Thus, the temporal/transient developments of the pulse provide us with a novel means of IR near-field optical coupling.

1. The strong reflection shown in Figure 2 can be used to focus IR radiation. This is the most common far-field optical coupling studied in literature.
2. The temporal/transient fields shown in Figure 3, that caused the strong diffraction, approximately 10% in Figure 2a, can be absorbed by placing the IR receiver close to the Au micropatch array plane, i.e., the near-field optical coupling.

## 5. Near-Field Optical Coupling and Phonon Band

**Figure 5:**
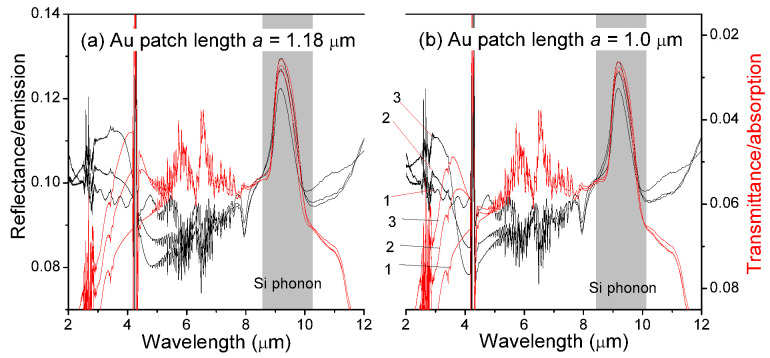
FTIR reflectance (left vertical axis, normal scale, black lines) and transmittance (right vertical axis, reversed scale, red lines). The Si phonon band is displayed as the emission in the reflectance spectrum and absorption in the transmittance spectrum. Line 1: SiO${}_{2}$/Si, Line 2: Au/Ti/SiO${}_{2}$/Si, $L_{y}=5.0$ μm, Line 3: Au/Ti/SiO${}_{2}$/Si, $L_{y}=2.5$ μm. (**a**) 1.18×0.24×0.05 μm${}^{3}$, $L_{x}=1.2$ μm, $L_{y}=5.0$ μm; 1.18×0.24×0.05 μm${}^{3}$, $L_{x}=1.1$ μm, $L_{y}=2.5$ μm. (**b**) 1.0×0.26×0.05 μm${}^{3}$, $L_{x}=1.22$ μm, $L_{y}=2.5$ μm; 1.0×0.26×0.05 μm${}^{3}$, $L_{x}=1.12$ μm, $L_{y}=5.0$ μm.

By the near-field optical coupling unraveled in the previous section, we can now understand the Si phonon band shown in Figure 2b. Figure 5 re-depict the experimental reflectance spectra (black lines) by the left vertical axis while the experimental transmittance spectra (red lines) by the right vertical axis with a reversed scale. Two Au/Ti/SiO${}_{2}$/Si samples are presented.

- Figure 5a Au/Ti micropatch length a=1.18μm, same in Figure 2b
- Figure 5b Au/Ti micropatch length a=1.1μm

revealing clearly that the designated Si photon band remained invariant of the Au/Ti micropatch structure. Two critical observations are

1. With the addition of the Au/Ti micropatch array, Si phonon emission (in the reflectance spectrum) and absorption (transmittance spectrum) are enhanced. And smaller $L_{y}$, i.e., larger surface metal coverage, caused larger enhancement. This is the result of the transient electromagnetic fields concentrated around the Au micropatches shown in Figure 3b,c, without which we would expect less stimulation to the phonons because
  - geometrical light rays would be blockaded by the added Au/Ti micropatches
  - larger reflection shown in Figure 2a,c would result in less penetration of stimulating light to Si phonons
2. The observed emission/absorption enhancement is accompanied by peak wavelength red-shift. This is one of the fundamental results of quantum scattering theory that a higher stimulation causes a larger red-shift (energy renormalization) in the emission and absorption spectra, see, e.g., [34].

## 6. Conclusions

Improving understanding of light-matter interaction is of pivotal importance for optoelectronics application. Metal microstrip arrays have been extensively studied as wavelength-selective optical coupling for IR sensing. In this work, spatial and temporal developments of far and near fields of NIR to LWIR radiations in a single layer of a two-dimensional gold micropatch array have been carefully studied by theoretical FDTD method and experimental FTIR study.

This work unravels quantitatively the link between the time-resolved microscopic dynamics of the electromagnetic field diffraction by the metal micropatch array and the experimentally observed far-field enhanced reflectance and reduced transmittance. Moreover, the diffracted field is shown to be transiently concentrated near the metal micropatch array.

We believe that this work will be very useful, providing new analyses of time-resolved FDTD study and opening up novel means of both far-field and near-field optical couplings.

## Data Availability

Not applicable.
